# The Influence of Mental Health on Job Satisfaction: Mediating Effect of Psychological Capital and Social Capital

**DOI:** 10.3389/fpubh.2022.797274

**Published:** 2022-02-08

**Authors:** Xin Cao, Heng Zhang, Peng Li, Xiaozhi Huang

**Affiliations:** ^1^School of Economics, Guangxi University, Nanning, China; ^2^School of Economics and Trade, Guangxi University of Finance and Economics, Nanning, China; ^3^School of Business Administration, Guangxi University, Nanning, China

**Keywords:** mental health, psychological capital, social capital, job satisfaction, mediating effect

## Abstract

Using data from the 2018 Chinese Family Panel Studies (CFPS), based on the mood-congruent theory, this study aims to explore the mechanisms of mental health on job satisfaction from the internal perspective (psychological capital) and external perspective (social capital). The results showed that (1) the two components of mental health have different effects on job satisfaction. The positive component of mental health had a positive effect on job satisfaction, while the negative component of mental health had a negative effect on job satisfaction; (2) Psychological capital and social capital play a mediating role in the relationship between mental health and job satisfaction. (3) After considering the potential endogenous problems between mental health and job satisfaction and conducting additional robustness analysis, including changing dependent variable and changing independent variable, our main results and influence mechanisms are remain robust and reliable. With the emergence of an increasingly competitive knowledge economy era, employees' mental health plays an important role in job satisfaction. Thus, it is imperative for managers to enhance employees' job satisfaction and better implement humanistic management by nurturing employees' psychological and social capital through the mental health.

## Introduction

Mental health is an important indicator of employees' psychological status, which directly affects their perception of job satisfaction (1). Managers are increasingly realizing that job satisfaction is the key to retaining excellent employees and organizational health (2–4). Therefore, companies must pay much attention to the employees' mental health conditions if they want to improve competitiveness. However, with the global economic slowdown, companies are facing an increasingly competitive environment (5).

The new changes in organizational environment are likely to cause new implications for the influencing mechanisms between mental health and job satisfaction. Moreover, competitive pressures outside the organization would translate into pressures for employees within the organization, which can be detrimental to mental health (6). This new change of organization environment and new challenges to employees have forced us to rethink the impact of mental health on job satisfaction, and the mechanisms in which mental health can affect job satisfaction is a theoretical question that needs to be answered urgently.

Mental health is a psychological state of living an active life (7), but poor mental health is often accompanied by a range of mental problems such as depression, anxiety, and fear (8). Therefore, mental health includes both positive and negative components (9–11). The positive component of mental health refers to individuals being in a positive state and hopeful about life, while the negative component of mental health refers to individuals being in a depressed and anxious state and living negatively (11, 12). Clearly, the two components of mental health have different effects on job satisfaction.

Current studies have confirmed that the correlation between mental health and job satisfaction (13). Generally, mental health exerts a positive effect on job satisfaction. Specifically, the positive component of mental health would induce positive emotions and employees can enjoy job satisfaction (14), while the negative component of mental health would induce negative emotions, which in turn reduces job satisfaction (15). However, with the changing of outside organizational environment, employees are facing new psychological challenges. In fact, the impact of the external competitive environment is detrimental to the mental health of employees (5). Meanwhile, mental health is closely connected with job satisfaction. Importantly, mental health includes all aspects of one's life (7, 11). Thus, mental health not only affects the internal psychological conditions of employees, but also influences their interaction with external environment. But it is unclear that how mental health would affect job satisfaction and more importantly, how to explain the relationship between mental health and job satisfaction from both internal and external aspects. In order to fill the gap of existing studies, based on the mood-congruent theory, we further explore the influence mechanisms between mental health and job satisfaction from both internal and external perspectives.

What are the avenues in which mental health can affect job satisfaction? According to mood-congruent theory, emotional states (positive and negative emotion) influence the way people remember, construct and evaluate target objects (16, 17). Generally, positive emotions activate positive information in the brain, causing positive evaluations and behaviors, while negative emotions focus on negative information, causing negative evaluations and behaviors. Thus, we argue that mental health exerts influences on individuals from both internal and external perspectives. On the one hand, from an internal perspective, mental health affects an individual's psychological capital, which in turn affects job satisfaction. As a positive psychological resource within employees, psychological capital is directly affected by mental health (18), and affects job satisfaction (19, 20). On the other hand, from an external perspective, mental health also affects social capital, which in turn affects job satisfaction. Social capital emphasizes the establishment of relationship network between individuals and others (21). In addition, mental health affects the individual's perception and evaluation of others (22–24), which in turn affects job satisfaction.

In summary, few studies have examined the mechanisms of the effect of mental health on job satisfaction from both internal and external perspectives, and have not yet answered whether mental health can influence job satisfaction from both internal perspective (psychological capital) and external perspective (social capital). Thus, this study hopes to answer the following questions: (1) whether the two components of mental health have different effects on job satisfaction; (2) whether psychological capital (internal perspective) and social capital (external perspective) mediate the effects of mental health on job satisfaction. Accordingly, using CFPS 2018 data, based on the mood-congruent theory, we test the effect of mental health on job satisfaction and further examine the mechanism of the influence of mental health on job satisfaction by introducing psychological capital from an internal perspective and social capital from an external perspective. Theoretically, we try to supplement the mechanism between mental health and job satisfaction to comprehensively understand the influence of mental health on employees and enrich the related literature on mental health and job satisfaction. Practically, we try to help employees recognize the importance of mental health and thus enhance their job satisfaction, as well as provide guidance for enterprises to maintain employees' mental health and improve their job satisfaction.

## Theory and Hypothesis

### Mental Health and Job Satisfaction

A person's state of health, includs not only physical health, but also, more importantly, mental health. Specifically, the World Health Organization's definition of health is a state of complete physical, mental and social well-being and not merely the absence of diseases or infirmity. Mental health is the psychological state of living an active life (25) and is a pleasurable experience. However, mental health includes all aspects of one's life (7, 26) and it is better to understand it from both positive emotional experiences and negative mental states. Job satisfaction reflects employees' attitudes and feelings about their jobs (27–31).

According to mind-congruent theory, positive emotions, which activate positive information in the brain, cause positive evaluations and behaviors, while negative emotions, which focus on negative information, cause negative evaluations and behaviors. Thus, the positive component of mental health usually refers to individuals in a state of pleasurable psychological experience (32), with positive emotions, which in turn increase the perception of job satisfaction. Conversely, the negative component of mental health refers to individuals in a depressive mental state, with negative emotions (12), which in turn decreases the perception of job satisfaction (33). Moreover, Li (34) reported that the positive component of mental health has a beneficial effect on job satisfaction, while the negative component (i.e., anxiety) of mental health has an adverse effect on satisfaction. Accordingly, we propose the following hypothesis:

H1: The positive component of mental health has a significant positive correlation with job satisfaction.
H2: The negative component of mental health has a significant negative correlation with job satisfaction.

### Psychological Capital and Job Satisfaction

With the development of positive psychology, psychological capital has been refined and widely used by scholars (21, 35, 36). Psychological capital is a crucial element of an individual's positive psychological state (37). As an emerging psychological resource, psychological capital can influence job satisfaction from an internal perspective (38). Generally, mood-congruent theory suggests that the more positive an employee's psychological state is, the higher his or her job satisfaction (17).

Psychological capital mainly includes four positive psychological factors: self-efficacy/confidence, hope, optimism and resilience (39). Employees who possess aboved psychological factors have the confidence to address challenges and finish tasks well and thus are more likely to perceive the meaning and value of jobs, leading to job satisfaction (4, 40). Specifically, resilience emphasizes individual compliance and behaving in accordance with organizational rules, so that employees can enjoy job satisfaction (39); self-efficacy/confidence, hope and optimism spur individual initiative and enthusiasm, allowing employees to fully explore their own potential (41). Employees who have enough psychological resources to complete job have high job satisfaction (42).

Furthermore, Ke et al. (43) divided psychological capital into task-oriented psychological capital and guanxi-oriented psychological capital and both of these elements were related to job satisfaction. Xu et al. (44) also found that nearly all dimensions of psychological capital (i.e., self-efficacy, hope, resilience, and optimism) were positively related to job satisfaction. Thus, the following hypothesis can be claimed:

H3: The psychological capital of employees has a significant positive correlation with their job satisfaction.

### Social Capital and Job Satisfaction

Social capital originated from the field of sociology and developed in different disciplines. However, to date, its definition has not been agreed upon among scholars. In fact, social capital mainly refers to the connections between individuals or groups and the resulting norms of reciprocity and trust, including social networks, social participation, and consensus of trust (45, 46). As an important social resource, social capital can affect employees' job satisfaction from an external perspective. First, employees can access work-related resources from their social networks (47), so that they can finish work better and achieve higher work performance, which leads to higher job satisfaction (48). In addition, employees can build relationships with trusted others in their social networks (49), share joys and solve problems, and improve job satisfaction through emotional interactions (39). Moreover, Stromgren et al. (50) found that social capital was positively associated with job satisfaction; Li and Xi (51) also found that social capital was an important factor in predicting job satisfaction. Accordingly, the following hypothesis is put forward:

H4: The social capital of employees has a significant positive correlation with their job satisfaction.

### Psychological Capital: The Mediating Role in the Link Between Mental Health and Job Satisfaction

The improvement of psychological capital depends on the development of positive psychological resources and the resolution of psychological problems (52). According to mind-congruent theory, positive psychology is beneficial to fully tap potential and enhance job ability of employees (53), thus increasing self-confidence, hope and resilience, which are the important components of psychological capital. Thus, the positive component of mental health is an positive psychological state, which can effectively nurture the individual's psychological capital (18) and enhance job satisfaction (44). However, in a negative mental state, individuals are susceptible to mental problems, which eventually leads to depletion of psychological resources (54). Thus, the negative component of mental health is harmful to the accumulation of psychological capital (55) and eventually leads to job dissatisfaction (42). Thus, we propose the following hypothesis:

H5: Psychological capital plays a mediating role in the relationship between mental health (positive component and negative component) and job satisfaction.

### Social Capital: The Mediating Role in the Link Between Mental Health and Job Satisfaction

The mental health status is closely related to social capital (49). According to mind-congruent theory, positive psychological status encourages employees to proactively interact with external surroundings and engage in extensive communication, emotional interaction with others (56), making it easier to build social networks and eventually promoting the accumulation of social capital. Therefore, the positive component of mental health is beneficial to the accumulation of social capital. Social capital allows employees to utilize relevant resources (57) to better carry out work and emotional expressions, thus enhancing job satisfaction. Conversely, a negative mental state induces employees to refuse to interact with their surroundings, communicate with people and build social networks, which is detrimental to the accumulation of social capital (58). Lacking of social capital makes it difficult for employees to have their needs met in finishing work and engaging in emotional interactions, which can trigger dissatisfaction with their jobs (59). Accordingly, the following hypotheses is made:

H6: Social capital plays a mediating role in the relationship between mental health (positive component and negative component) and job satisfaction.

### Theoretical Model

**Figure 1:**
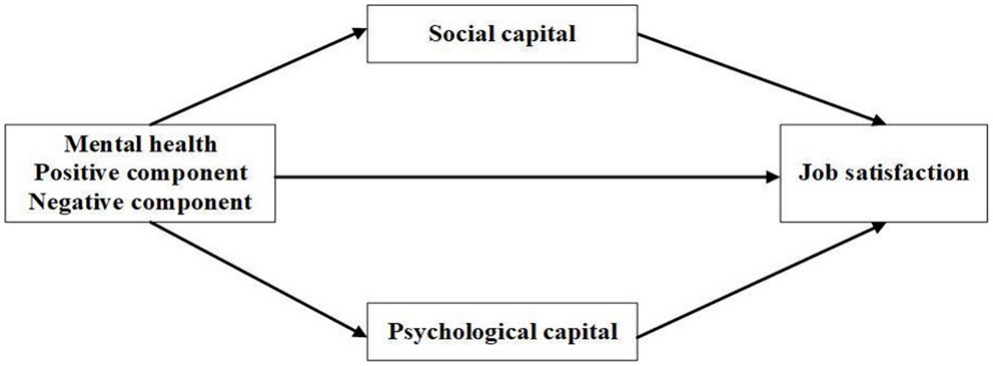
The theoretical model.

Based on the mood-congruent theory, this study examine the effect of different components of mental health on job satisfaction. In order to better unveil the theoretical “black box” of mental health on job satisfaction, we introduce psychological capital from the internal perspective and social capital from the external perspective to deeply explore the mechanism between mental health and job satisfaction. Thus, a theoretical model is constructed (Figure 1).

## Materials and Methods

### Research Data

This study used the Wave 5 (2018) survey of the China Family Panel Studies (CFPS), which was initiated by Peking University. The respondents of this survey are Chinese residents. The baseline survey of CFPS was initiated in 2010 and followed by four rounds of tracking surveys in 2012, 2014, 2016, and 2018, with samples covering 25 provinces/cities/autonomous regions in China (57). After excluding observations with missing data and respondents with no income, the final sample was restricted to 6,741 observations.

### Variables and Measurements

#### Mental Health

The Epidemiologic Studies Depression Scale developed by Radloff (60) in CFPS, includs positive and negative components. We refer to the method of Xi (61), using the simplified CES-D scales to measure employees' mental health, and Turvey et al. (62), who confirmed that this scale has good reliability and validity. Specifically, the respondents reported the rate of feelings or behaviors in the past week, such as “I am pleasured with my life,” “I feel happy,” “I feel depressed,” “I feel sad,” “I feel upset,” “I find it hard to do anything,” “I feel like life can't go on,” “I do not sleep well.” The scale ranged from 1(few) to 4 (mostly). Then we merged the first two responses into a new indicator, namely pm (Cronbach's α = 0.75) to represent the positive component of mental health and the last six responses into a new indicator, namely nm (Cronbach's α = 0.76), to represent the negative component of mental health.

#### Psychological Capital

This study refers to the method of Liu et al. (63), using the response of “I am confident about future” as the proxy variable for psychological capital. The responses ranged from 1(strongly disagree) to 5 (strongly agree).

#### Social Capital

We refer to the method of Jiao and Chen (64), using social trust as the proxy variable for social capital.These scale items are as follows: “trust in parents,” “trust in neighbors,” “trust in Americans,” “trust in strangers,” “trust in local government officials,” and “trust in doctors.” It is an 11-point scales (Cronbach's α = 0.68), ranging from 0 (strongly distrust) to 10 (strongly trust).

#### Job Satisfaction

Job satisfaction is measured by 4 responses: “job income satisfaction,” “job safety satisfaction,” “job environment satisfaction,” and “job time satisfaction,” using a 5-point scales (Cronbach's α = 0.75), ranging from 1(strongly disagree) to 5 (strongly agree).

#### General Job Satisfaction

We use the response item of “general job satisfaction” to measure general job satisfaction, from 1(strongly disagree) to 5(strongly agree).

To summarize, the details of the variables are shown in Table 1.

**Table 1:** Variable names and definitions.

**Variables**	**Name**	**Index**	**Definition**
Explained variables	Worksatisfy	1–5	Job satisfaction
	worktt	1–5	General job satisfaction 1 = strongly disagree, 5 = strongly agree
Explanatory variables	pm	1–4	Positive component of mental health
	nm	1–4	Negative component of mental health
Mediator variable	pc	1–5	Psychological capital
	sc	0–10	Social capital
Control variables	Age	16–96	Age
	Account	0, 1	Account, 1 = agricultural household 0 = Non-agricultural household
	Marriage	0, 1	Marriage, 1 = married 0 = unmarried and else
	Familysize	1–17	Number of family members
	Educ	0, 1	Education,1 = above senior high school 0 = senior high school and below
	Income	1–500,000	Job income
	Lnincome	0–13.12	Logarithm of job income
	Health	0, 1	Physical health, 1 = healthy, 0 = unhealthy
Instrumental variable	Sex	0, 1	Sex, 1 = male, 0 = female
	East	0, 1	Region, 1 = east, 0 = Middle west

### Analytical Strategy

Using R 4.1.0 for data analysis. First, we employed OLS regressions to test Hypotheses 1 to 4, respectively. Second, considering the potential endogenous problems between mental health and job satisfaction, we use instrument variable to resolve it. Third, we further conduct additional robustness analysis, including changing dependent variable and changing independent variable to enhance robustness of results.

## Results

### Descriptive Statistical Analysis

As is shown in Table 2, Job satisfaction averaged 3.57 and the mean value of general job satisfaction is 3.61, which verifies that the level of job satisfaction of employees is not very high. The positive component of mental health averaged 2.96 and the negative component of mental health averaged 1.54, both indicating that employees have a high level of mental health; The psychological capital averaged 4.16 and the social capital averaged 5.55, indicating that employees' psychological capital and social capital was slightly higher.

**Table 2:** Descriptive statistics (*N* = 6,741).

**Variables**	**Mean**	**SD**	**Min**	**Max**
Worksatisfy	3.57	0.75	1	5
worktt	3.61	0.86	1	5
pm	2.96	0.77	1	4
nm	1.54	0.45	1	4
pc	4.16	0.85	1	5
sc	5.55	1.32	0	10
Age	32.78	8.57	16	96
Marriage	0.66	0.47	0	1
Familysize	3.98	2.13	1	17
Account	0.68	0.47	0	1
Educ	0.54	0.50	0	1
Income	41,965.33	35,952.29	1	500,000
Inincome	10.30	0.94	0	13.12
Health	0.86	0.35	0	1
Sex	0.57	0.50	0	1
East	0.48	0.50	0	1

*Worksatisfy, job satisfaction; worktt, general job satisfaction; pm, positive component of mental health;nm, negative component of mental health; pc, psychological capital; sc,social capital; educ, education; health, physical health*.

### OLS Regression

Table 3 shows the results from the OLS regressions. As indicated in Model (1), male and agricultural accounts showed lower levels of job satisfaction, while physical health and higher levels of education showed higher levels of job satisfaction. Income level is beneficial to maintain job satisfaction, whereby a 1%-unit increase in income level resulted in a ~0.0007-unit increase in job satisfaction. Additionally, the effect of age on job satisfaction was a positive U-shape. This means that job satisfaction is higher at younger ages, then gradually decreases, to its lowest point at age 26, and then gradually rises again.

**Table 3:** The two components of mental health regressed on job satisfaction.

	**(1)**	**(2)**	**(3)**	**(4)**	**(5)**	**(6)**
pm		0.13^***^		0.08^***^	0.07^***^	0.06^***^
		(0.01)		(0.01)	(0.01)	(0.01)
nm			−0.24^***^	−0.17^***^	−0.17^***^	−0.15^***^
			(0.02)	(0.02)	(0.02)	(0.02)
sc				0.07^***^		0.07^***^
				(0.01)		(0.01)
pc					0.11^***^	0.10^***^
					(0.01)	(0.01)
Health	0.28^***^	0.24^***^	0.22^***^	0.19^***^	0.18^***^	0.17^***^
	(0.03)	(0.03)	(0.03)	(0.03)	(0.03)	(0.03)
Sex	−0.23^***^	−0.22^***^	−0.25^***^	−0.24^***^	−0.25^***^	−0.24^***^
	(0.02)	(0.02)	(0.02)	(0.02)	(0.02)	(0.02)
Account	−0.08^***^	−0.07^***^	−0.07^**^	−0.07^**^	−0.07^**^	−0.07
	(0.02)	(0.02)	(0.02)	(0.02)	(0.02)	(0.02)
Marriage	0.01	0.002	0.01	0.01	−0.01	0.002
	(0.03)	(0.03)	(0.03)	(0.02)	(0.02)	(0.02)
Age	−0.05^***^	−0.05^***^	−0.05^***^	−0.04^***^	−0.05^***^	−0.04^***^
	(0.01)	(0.009)	(0.01)	(0.01)	(0.01)	(0.01)
Age∧2	0.001^***^	0.001^***^	0.001^***^	0.001^***^	0.001^***^	0.001^***^
	(0.0001)	(0.0001)	(0.0001)	(0.0001)	(0.0001)	(0.0001)
Educ	0.13^***^	0.12^***^	0.11^***^	0.08^***^	0.12^***^	0.09^***^
	(0.02)	(0.029)	(0.02)	(0.02)	(0.02)	(0.02)
Familysize	−0.003	−0.004	−0.01	−0.01	−0.01^**^	−0.01^**^
	(0.004)	(0.004)	(0.004)	(0.004)	(0.004)	(0.004)
Lnincome	0.07^***^	0.06^***^	0.06^***^	0.05^***^	0.06^***^	0.05^***^
	(0.01)	(0.01)	(0.01)	(0.01)	(0.01)	(0.01)
Constant	3.65^***^	3.29^***^	4.12^***^	3.42^***^	3.35^***^	3.10^***^
	(0.15)	(0.15)	(0.15)	(0.16)	(0.16)	(0.17)
*N*	6,741	6,741	6,741	6,741	6,741	6,741
*R^2^*	0.07	0.09	0.09	0.11	0.11	0.12

Robust standard errors in parentheses, *p < 0.1,

** *p < 0.05*,

*** *p < 0.01. pm, positive component of mental health; nm, negative component of mental health; pc, psychological capital; sc, social capital; health, physical health; educ, education*.

To continue, in models 2 and 4–6, after adding the control variables, the positive component of mental health has a positive correlation with job satisfaction, whereby a 1-unit increase in positive component of mental health resulted in a 0.06 ~ 0.13-unit increase in job satisfaction. Conversely, from Model (3) to (6), with inclusion of the control variables, the negative component of mental health has a negative correlation with job satisfaction, whereby a 1-unit increase in positive component of mental health resulted in a 0.15 ~ 0.24-unit drop in job satisfaction. Our Hypothesis 1 and Hypothesis 2 are supported.

Moreover, Model (4) presents the positive effects of social capital on job satisfaction, whereby a 1-unit increase in social capital resulted in a 0.07-unit increase in job satisfaction and Model (5) presents the positive effects of psychological capital on job satisfaction, whereby a 1-unit increase in psychological capital resulted in a 0.11-unit increase in job satisfaction. Hypothesis 3 and Hypothesis 4 are supported.

### Instrumental Variable Regression

Given the potential bidirectional causality between mental health and job satisfaction, this study employs instrumental variable to reduce endogeneity. We refer to the method of Zheng (65) and use regional dummy variables as instrumental variable. Because Yu et al. (32) theoretically revealed that temperature can affect mental health, thus this study uses region as a proxy variable for temperature. First, regional differences in temperature cause changes in mental health (66), meeting the correlation between independent variable and instrumental variable. In addition, regional differences in temperature do not directly affecting job satisfaction, meeting the exogeneity. Furthermore, considering the difference in temperature between eastern and midwestern regions of China (67), the impact of temperature on mental health is also different. Therefore, the eastern region is chosen as instrumental variable.

The results of the statistical tests indicate that the selected instrumental variable was not weak (*p* < 0.05) and the results of the Wu-Hausman test indicat that the results of regressions of instrumental variable were superior to those of OLS (*p* < 0.01). Additionally, we can found that the coefficients of the two components of mental health passed the Wald test (*p* < 0.01). This means that the positive component of mental health has a significant positive correlation with job satisfaction, while the negative component of mental health has a significant negative correlation with job satisfaction (See Table 4).

**Table 4:** The results of instrumental regression.

**Dependent variable**	**Job satisfaction**			
	**(1)**	**(2)**	**(3)**	**(4)**
pm	2.10^***^	2.10^**^		
	(0.75)	(1.05)		
nm			−2.08^***^	−1.73^***^
			(0.50)	(0.51)
Health		−0.35		−0.11
		(0.32)		(0.12)
Sex		−0.05		−0.34^***^
		(0.10)		(0.04)
Account		0.05		−0.02
		(0.07)		(0.03)
Marriage		−0.18^**^		−0.09^***^
		(0.08)		(0.03)
Age		0.002		0.001
		(0.003)		(0.002)
Educ		−0.07		0.02
		(0.11)		(0.04)
Familysize		−0.02		−0.03^***^
		(0.01)		(0.01)
Lnincome		0.01		0.01
		(0.03)		(0.02)
Constant	−2.63	−2.26	6.76^***^	6.55^***^
	(2.23)	(2.60)	(0.76)	(1.09)
*N*	6,741	6,741	6,741	6,741
Adj *R^2^*	−3.86	−3.85	−1.16	−0.68

Robust standard errors in parentheses, *p < 0.1,

** *p < 0.05*,

*** *p < 0.01. pm, positive component of mental health; nm, negative component of mental health; health, physical health; educ, education*.

In conclusion, the results are remain robust when employing instrumental variable to address the endogeneity problem.

### Mechanism Analysis

**Figure 2:**
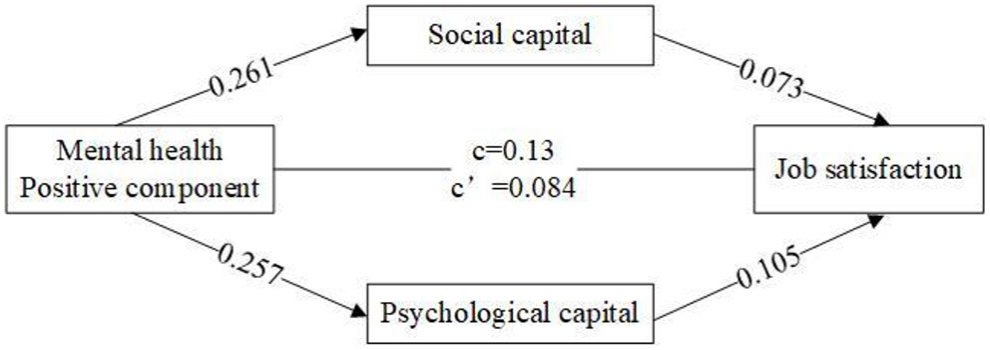
The mediating role of psychological and social capital between the positive component of mental health and job satisfaction.

We employed bootstrapping methods (68) to test whether the indirect effects were statistically significant (5,000 resamples). The significance of indirect effects depends on whether the 95% confidence intervals include zero. The results showed that the mediating effect of social capital between the positive component of mental health and job satisfaction was significant with a 95% confidence interval of (0.015, 0.024), excluding 0, and an indirect effect value of 0.019; meanwhile, the mediating effect of psychological capital between the positive component of mental health and job satisfaction was significant with a 95% confidence interval of (0.021, 0.034), excluding 0, and an indirect effect value of 0.027. As a result, social capital and psychological capital play a mediating role between the positive component of mental health and job satisfaction (see Figure 2).

**Figure 3:**
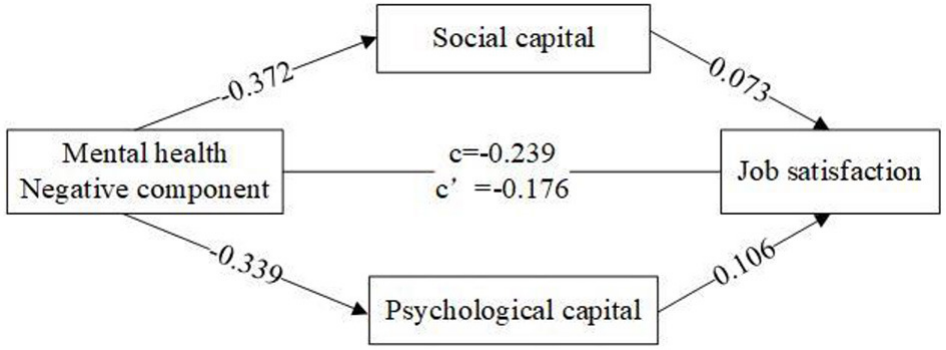
The mediating role of psychological and social capital between the negative component of mental health and job satisfaction.

Similarly, we found that the mediating effect of social capital between the negative component of mental health and job satisfaction was significant, with a 95% confidence interval of (−0.035, −0.02), excluding 0, and indirect effect value of −0.027. The mediating effect of psychological capital between the negative component of mental health and job satisfaction was significant, with a 95% confidence interval of (−0.046, −0.027), excluding 0, and an indirect effect value of −0.036. Thus, we can conclude that social capital and psychological capital play a mediating role between the negative component of mental health and job satisfaction (see Figure 3).

Additionally, we perform the mechanisms using the method of regressions and bootstrapping. We can found that the positive component of mental health had positive effect on psychological capital in Model (3) and social capital in Model (2). Moreover, we also noticed that the influence of the positive component of mental health on job satisfaction in Model (1) tended to diminish in Model (4), and its coefficient changed from 0.13 to 0.08. This implies the link between positive component of mental health and job satisfaction was partially mediated by social capital and psychological capital (see Supplementary Table 1). Supplementary Tables 2, 3 also indicated the confidence interval of indirect effect of psychological capital and social capital, excluding 0. Similarly, we also found that the link between negative component of mental health and job satisfaction was partially mediated by the indicators of social capital and psychological capital (see Supplementary Tables 4–6).

In summary, this study verified the mediating role of psychological and social capital in the relationship between the two components of mental health and job satisfaction, thus supporting H5 and H6.

## Robustness Analysis

### Changing the Dependent Variable

We use the general job satisfaction to verify main results again.

**Figure 4:**
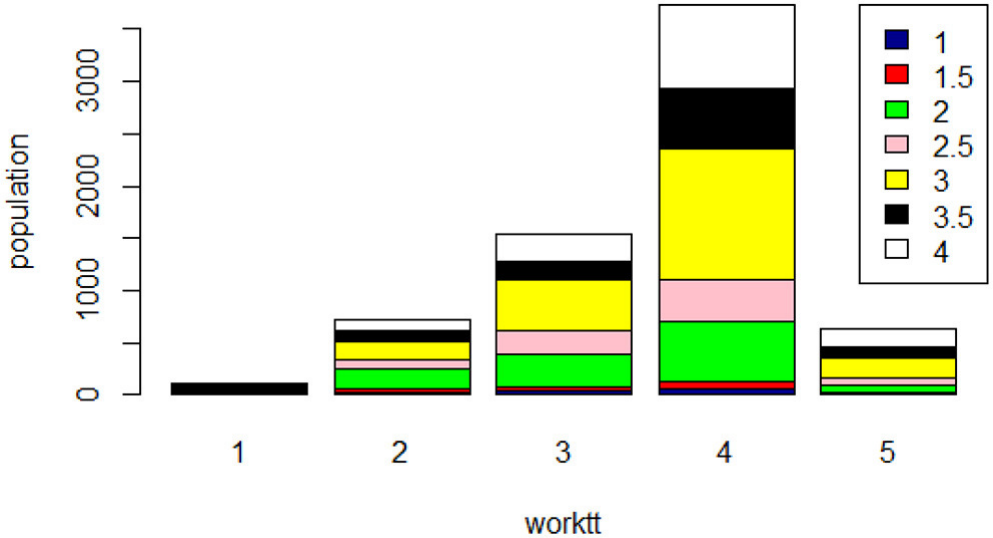
The relationship between mental health and general job satisfaction (worktt is the general job satisfaction; population is the number of employee).

First, we found that the positive componet of mental health is closely connected with job satisfaction. In Figure 4, general job satisfaction ranges from 1 (strongly dissatisfied) to 5 (strongly satisfied), with larger values indicating higher job satisfaction. The upper right side of the figure is the positive component of mental health, with larger values indicating higher mental health. Obviously, more psychologically healthy employees have a larger percentage of general job satisfaction. Thus, we can conclude that the mental health of employees directly affects job satisfaction (69) (see Supplementary Figure R1).

### Ordered Probit Regression

Considering that general job satisfaction is an ordered variable, we employed ordered probit model to perform the analysis. Table 5 shows the optimized probit regression model. Apparently, the two components of mental health (positive vs. negative), psychological capital, and social capital have significant effects on general job satisfaction. This means that Hypotheses 1 to 4 are supported again. Specifically, the positive component of mental health positively affects job satisfaction with an OR of 1.12, indicating that controlling for other variables, a 1-unit increase in the positive component of mental health resulted in 1.12 times increase in the level of satisfaction, while the negative component of mental health negatively affects job satisfaction with an OR of 0.86, indicating that controlling for other variables, a one-unit increase in the negative component of mental health resulted in 0.86 times increase in the level of satisfaction; Social capital positively affects job satisfaction with an OR of 1.11, indicating that controlling for other variables, a one-unit increase in social capital resulted in 1.11 times increase in the level of satisfaction; psychological capital negatively affects job satisfaction with an OR of 1.17, indicating that controlling for other variables, a one-unit increase in the psychological capital resulted in 1.17 times increase in the level of satisfaction.

**Table 5:** The results of ordered probit regression.

		**β**	**S.E**	**OR**	***P***	**95% CI**	
						**Lower**	**Upper**
Valve value	[worktt = 1]	−0.56	0.14	0.57	0.00	0.43	0.75
	[worktt = 2]	0.49	0.13	1.63	0.00	1.26	2.13
	[worktt = 3]	1.31	0.14	3.71	0.00	2.84	4.84
	[worktt = 4]	3.08	0.14	21.78	0.00	16.60	28.59
Position	pm	0.12	0.02	1.12	0.00	1.08	1.17
	nm	−0.16	0.03	0.86	0.00	0.81	0.91
	sc	0.11	0.01	1.11	0.00	1.09	1.14
	pc	0.16	0.02	1.17	0.00	1.14	1.22
	Health	0.23	0.04	1.25	0.00	1.16	1.35
	Age	0.003	0.002	1.003	0.06	1.00	1.006
	Educ	0.06	0.03	1.06	0.03	1.01	1.12

*Worktt, general job satisfaction; pm, positive component of mental health; nm, negative component of mental health; pc, psychological capital; sc, social capital; educ, education; health, physical health*.

Importantly, our model passed the likelihood ratio test (*p* < 0.01), indicating that the model is fully valid and well-fitted.

### Changing the Independent Variable

We combined the eight scale into a single indicator to represent the mental health (1). Specifically, we reverse-coded items of the positive component of mental health to obtain a general indicator of mental health, namely mental (Cronbach's α = 0.75). The high score means the poor mental health.

### OLS Regression

As shown in Supplementary Table R1, after adding control variables, the mental health has a negative correlation with job satisfaction, whereby a 1-unit increase in mental health resulted in a 0.21 ~ 0.29-unit drop in job satisfaction. The effect of mental health on job satisfaction was supported. Moreover, Model (3) presents the positive effects of social capital on job satisfaction and Model (4) presents the positive effects of psychological capital on job satisfaction. Hypothesis 3 and Hypothesis 4 are partially supported.

### Instrumental Variable Regression

Supplementary Table R2 reports the results of instrumental variable regression. We can found that the coefficients of the mental health passed the Wald test (*p* < 0.01). This means that the mental health has a negative influence on job satisfaction. The results are robust as above.

### Mechanism Analysis

In Supplementary Table R3, we can found that mental health had negative effect on psychological capital in Model (3) and social capital in Model (2). Moreover, we also noticed that the influence of mental health on job satisfaction in Model (1) tended to diminish in Model (4) and its absolute value of coefficient changed from 0.30 to 0.22. This implies the link between mental health and job satisfaction was partially mediated by social capital and psychological capital. What's more, we found that the mediating effect of social capital between the mental health and job satisfaction was significant, with a 95% confidence interval of (−0.04, −0.03), excluding 0, and indirect effect value of −0.04. The mediating effect of psychological capital between mental health and job satisfaction was significant, with a 95% confidence interval of (−0.06, −0.04), excluding 0, and an indirect effect value of −0.05 (see Supplementary Tables R4, R5). Thus, social capital and psychological capital play a mediating role between mental health and job satisfaction.

To sum up, our results are very robust and reliable.

## Discussion

With the development of society, mental health is becoming more and more important to maintain happy life and job satisfaction (22). Unfortunately, the mental health of Chinese residents is not optimistic and many people are suffering from the poor mental health (70). Based on the mood-congruent theory, this study examined the underlying mechanisms between the effect of mental health on job satisfaction in the Chinese settings. We showed that the two components of mental health significantly affected job satisfaction. The negative component of mental health has an adverse on job satisfaction. While the positive component of mental health exerts a positive effect on job satisfaction. Our results is accordance with the recent studies. Warszewska-Makuch (1) found that the negative component of mental health negatively predicted job satisfaction in western countries. Zhang et al. (13) used different samples from China and Iran to verify that the negative component of mental health is detrimental to job satisfaction. To some extent, the effect of mental health condition on job satisfaction is robust. Therefore, managers should emphasis on importance of mental health and help employees to mitigate the adverse effects of poor mental health.

Furthermore, current studies have verified mental health can exert effect on psychological capital (18, 53, 54) and social capital (49, 58); Meanwhile, psychological capital (4, 39) and social capital (49, 59) have a positive effect on job satisfaction, but lacked an integrate perspective to better understand the effect of mental health on job satisfaction. Therefore, we further fill the gaps between mental health and job satisfaction. Specifically, mental health not only affects the accumulation of internal psychological capital, but also the establishment of external relationships between employees and others, which in turn affects job satisfaction.

As intangible capital, social capital and psychological capital is playing an important role. Social capital and psychological capital exert influences on entrepreneurial performance (14), entrepreneurial intentions (71), quality of life (72), and second victim severity (73). Therefore, future research can explore more from the dual perspectives of psychological capital and social capital, such as job performance and job burnout (74, 75).

Actually, our study may provide a new perspective to better understand the influence factors of mental health. From an internal perspective, organization's information-providing behaviors can affect mental health through cognitive and affective aspects (5). From an external perspective, labor values affect mental health through social support (22). Therefore, future research could examine the influence factors or consequences of mental health from dual perspectives.

Additionally, in terms of individual differences, we show that males had lower levels of job satisfaction. This may be attributed to males suffering from more interpersonal pressure and work content pressure (34); we also found that employees in rural households have lower job satisfaction, which may because the social capital among them is lower than urban employee (76) while physical health, higher incomes and high school education or more were indicators of higher levels of job satisfaction. This because physical health, high incomes, and high education level will induce positive emotions and obtain more psychological and social resources to finish job better (77). Furthermore, the effect of age on job satisfaction was a positive U-shape. In young age, the ability and skill of job is insufficient to wrestle with pressure and as they get older, employees become more comfortable with their jobs. Accordingly, when managers try to maintain mental health of employees, they should consider the individual difference.

To sum up, the mental health is a prerequisite for organizational health and sustainability (5). It is high time for enterprises to pay much attention to the mental health of employees.

### Theoretical Contributions

First, this research contributes to the literature on mental health. We examine the impact of mental health from both positive positive component and negative component. We explored the different effects of the two components of mental health with the aim of improving mental health in a targeted manner.

Moreover, based on mood-congruent theory, from the internal and external perspectives, we comprehensively examined the impact of mental health on employees. On the one hand, from internal perspective, the positive component of mental health has a positive effect on psychological capital; meanwhile, the negative component of mental health has a negative effect on psychological capital. On the other, from external perspective, the positive component of mental health has a positive effect on social capital; meanwhile, the negative component of mental health has a negative effect on social capital. Our results contributed to a better understanding of mental health.

Second, this research contributes to the literature on job satisfaction. Job satisfaction reflects employees' positive perception of job, and studies have extensively explored the factors that influence job satisfaction (12, 34, 40, 44, 78). Based on mood-congruent theory, from both internal and external aspects, the current work introduce psychological capital and social capital to further reveals the mechanism of the effect of mental health on job satisfaction and answers the question of how the two components of mental health affect job satisfaction and the individual difference of job satisfaction. Undoubtedly, the findings of this study expand on the literature by exploring the factors influencing job satisfaction from the psychological level and social perspectives, thus providing a better understanding of job satisfaction.

Third, this research theoretically extends the application of psychological capital. As an important psychological resource, psychological capital has been widely focused (37, 38, 42, 53, 54). From the internal perspective, we found that the conditions of mental health exert influence on psychological capital and we showed that the two components of mental health affect job satisfaction through psychological capital and extended the application of psychological capital as a mediating mechanism.

Importantly, our study clarifies the relationship between mental health, psychological capital and job satisfaction and confirms the indirect role of psychological capital between mental health and job satisfaction at the psychological level, which is conducive to better understanding of the positive “soft” impact of psychological capital.

Finally, this research theoretically extends the application scenario of social capital. As an important social resource for employees (57), social capital is an important factor affecting job satisfaction (50). From the external perspective, we found that mental health have effect on the social capital and we further found that social capital is a bridge between mental health and job satisfaction. This will not only help clarify the relationship between mental health, social capital and job satisfaction, but also extend the application scenarios of the “soft” functions of social capital.

### Practical Implications

Actually, our results are enlightening for managers to manage the mental health of employees under the COVID-19 pandemic. Since the outbreak of COVID-19, people's daily lives have fundamentally changed (79). Under the threat of the pandemic, it is plausible that individuals' mental health has been affected (79–82). During the COVID-19 outbreak, Zhu et al. (81) conducted a survey on mental health status of Chinese employees and found that 30.8% of employees experienced significant stress responses, 51.3% experienced mild to moderate depressive responses, and 43.3% experienced mild to moderate anxiety responses. Similarly, most people experienced psychological problems (i.e., anxiety, stress, depression) (79, 83) and have a tendency to suicide (84) under the COVID-19.

How to recovery and manage mental health from the COVID-19 pandemic is an important question (85). McDaid (85) suggest that actions should be taken to cope with the adverse effect of mental ill health for policy makers, such as developing long term mental health support plans and social protection policies; Additionally, psychiatrists should shoulder responsibilities to care for patients during the COVID-19 pandemic (86); To continue, suicide caused by the COVID-19 pandemic should be taken seriously and strategies should be adopted to prevent it (84). Similarly, managers also should to manage and recovery the mental health of employees from the COVID-19 pandemic. Therefore, according to our results, it is crucial for enterprises to alleviate the adverse effects caused by employees' mental problems.

Moreover, according to our results, we make the following suggestions: (1) On one hand, based on mood-congruent theory, employers should give attention to employees' mental health and enhance their positive emotional experiences. The positive component of mental health can directly improve job satisfaction. On the other hand, giving close attention to employees' negative mental states, reduces their negative impact on job satisfaction. Enterprises should establish an employee mental health assessment mechanism to give close attention to employees' mental health status and prevent the negative impact of poor mental health. (2) Organizations can improve employees' initiative from the internal perspective of psychological capital accumulation. Specifically, providing mental health training courses and teaching psychological capital accumulation skills are useful ways; In addition, employers should know how to value employees, by emphasizing a human-oriented view of talent, enhancing employees' emphasis and self-confidence is also good ways; (3) From the external perspective, enterprises should pay attention to the accumulation of employees' social capital by building employee interaction platforms, sharing information and resources, and helping employees build social networks; On the other, employees ought to take the initiative to interact with the world, continuously expand social circles, and make full use of the resources in social networks to improve job performance and enjoy emotional expression. (4) It is important for organizations to consider individual differences when it comes to job satisfaction. Considering employees' gender, age, education, household registration, income, and physical health is meaningful in enhancing job satisfaction.

### Limitations and Future Research

First, this research is conducted in Chinese culture background, resulting in the generalizability of results being limited to Chinese context. Given the difference of cross-culture, is there a possible difference between the mechanism of mental health on job satisfaction and the research based on Chinese samples? The future research can verify the above conclusions through cross-cultural comparative analysis. Second, although we examined the influence mechanisms between mental health and job satisfaction from internal (psychological capital) and external (social capital) paths, we did not discuss the relationship between psychological capital and social capital. So future research can further discuss the relationship between psychological and social capital. Third, although we explore the mechanisms of mental health on job satisfaction, however we does not explore its boundary conditions. Therefore, future research can explore the boundary conditions of mental health on job satisfaction. Finally, our study mainly examines individual-level variables and does not consider family-level factors on job satisfaction. The boundary between family and work is increasingly blurred in current society, thus future research can start at the family level (work-family conflict, work-family gain) to further examine the mechanisms and boundary conditions of mental health on job satisfaction.

## Conclusion

In summary, based on the mood-congruent theory, this study examined the effect of different components of mental health on job satisfaction in Chinese settings. We further explore the mechanism of mental health on job satisfaction from the internal perspective (psychological capital) and external perspective (social capital). The results showed that the different components of mental health had different effects on job satisfaction and furthermore, psychological capital and social capital play a mediating role in the relationship between mental health and job satisfaction. Importantly, after considering the potential endogenous problems between mental health and job satisfaction and conducting additional robustness analysis, including changing dependent variable and changing independent variable, our main results and influence mechanisms are remaining robust and reliable. Therefore, in order to promote job satisfaction of employees through the mental health, we call for not only improvement in psychological capital from the internal perspective, but also social capital for them from the external perspective.

## Data Availability Statement

Publicly available datasets were analyzed in this study. This data can be found at: http://www.isss.pku.edu.cn/cfps/download.

## Ethics Statement

The studies involving human participants were reviewed and approved by Peking University. The patients/participants provided their written informed consent to participate in this study.

## Author Contributions

XC organized the database, performed data analysis and wrote the revised version. HZ performed statistical analysis and wrote the first draft of manuscript. PL added robustness analysis and wrote the revised version. XH contributed to conceptualization and methodology. All authors contributed to the article and approved the submitted version.

## Funding

This work was supported by the National Social Science Key Foundation of China under Grant No. (17AJL012), the National Natural Science Foundation of China under Grant Nos. (71872055 and 72062001), and Innovation Project of Guangxi Graduate Education under Grant No. YCSW2021058.

## Conflict of Interest

The authors declare that the research was conducted in the absence of any commercial or financial relationships that could be construed as a potential conflict of interest.

## Publisher's Note

All claims expressed in this article are solely those of the authors and do not necessarily represent those of their affiliated organizations, or those of the publisher, the editors and the reviewers. Any product that may be evaluated in this article, or claim that may be made by its manufacturer, is not guaranteed or endorsed by the publisher.
