# Supplementary material for: The Influence of Mental Health on Job Satisfaction: Mediating Effect of Psychological Capital and Social Capital
Source: Front Public Health. 2022 Feb 8;10:797274. doi: 10.3389/fpubh.2022.797274 (PMC8860985; doi:10.3389/fpubh.2022.797274)
Supplement: Supplementary file 2 [file Data_Sheet_2.docx]

Supplementary Material

# Supplementary Data

This data can be found at: http://www.isss.pku.edu.cn/cfps/download.

# Supplementary Figures and Tables

## Supplementary Figure


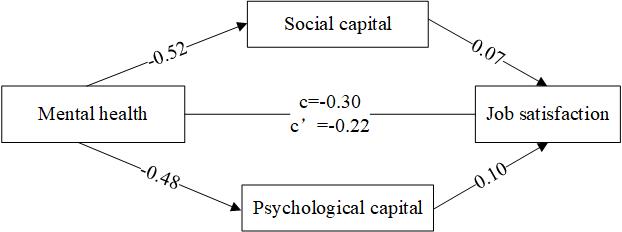


**FIGURE R1 |** The mediating role of psychological and social capital between mental health and job satisfaction.

## Supplementary Tables

**Alternative mechanism Analysis**

We again use the R package of BruceR to test the mechanism between mental health and job satisfaction.

**TABLE 1 |** The method of regression (1)

|  | (1) worksatisfy | (2) sc | (3) pc | (4) worksatisfy |
| --- | --- | --- | --- | --- |
| (intercept) | 3.57*** | 5.55*** | 4.16*** | 3.57*** |
|  | (0.01) | (0.02) | (0.01) | (0.01) |
| health | 0.25*** | 0.28*** | 0.27*** | 0.20*** |
|  | (0.03) | (0.05) | (0.03) | (0.03) |
| sex | -0.21*** | 0.01 | 0.09*** | -0.22*** |
|  | (0.02) | (0.03) | (0.02) | (0.02) |
| account | -0.06** | 0.03 | 0.05* | -0.06** |
|  | (0.02) | (0.04) | (0.02) | (0.02) |
| age | 0.00 | -0.006*** | -0.001 | 0.00 |
|  | (0.001) | (0.002) | (0.001) | (0.001) |
| educ | 0.12*** | 0.41*** | -0.09*** | 0.10*** |
|  | (0.02) | (0.04) | (0.02) | (0.02) |
| familysize | -0.01* | -0.01 | 0.03*** | -0.01** |
|  | (0.004) | (0.01) | (0.01) | (0.004) |
| lnincome | 0.04*** | 0.08*** | 0.02 | 0.03*** |
|  | (0.01) | (0.02) | (0.01) | (0.01) |
| pm | 0.13*** | 0.26*** | 0.26*** | 0.08*** |
|  | (0.01) | (0.02) | (0.01) | (0.01) |
| sc |  |  |  | 0.07*** |
|  |  |  |  | (0.01) |
| pc |  |  |  | 0.11*** |
|  |  |  |  | (0.01) |
| R^2 | 0.08 | 0.08 | 0.08 | 0.11 |
| Adj. R^2 | 0.08 | 0.07 | 0.08 | 0.11 |

*p<0.1; **p<0.05; ***p<0.01. pm, positive component of mental health;nm, negative component of mental health;pc, psychological capital; sc, social capital; educ, education; health, physical health.

**TABLE 2 |** Indirect path: "pm" (X) ==> "sc" (M) ==> "worksatisfy" (Y)

|  | Effect | S.E | z | p | [Boot 95% CI] |
| --- | --- | --- | --- | --- | --- |
| Indirect (ab) | 0.019 | 0.002 | 7.907 | <0.001*** | [0.015, 0.024] |
| Direct (c') | 0.084 | 0.012 | 6.817 | <0.001*** | [0.059, 0.107] |

**TABLE 3 |** Indirect path: "pm" (X) ==> "pc" (M) ==> "worksatisfy" (Y)

|  | Effect | S.E | z | p | [Boot 95% CI] |
| --- | --- | --- | --- | --- | --- |
| Indirect (ab) | 0.027 | 0.003 | 8.290 | <0.001*** | [0.021, 0.034] |
| Direct (c') | 0.084 | 0.012 | 6.817 | <0.001*** | [0.059, 0.107] |

**TABLE 4 |** The method of regression (2)

|  | (1) worksatisfy | (2) sc | (3) pc | (4) worksatisfy |
| --- | --- | --- | --- | --- |
| (intercept) | 3.57*** | 5.55*** | 4.16*** | 3.57*** |
|  | (0.01) | (0.02) | (0.01) | (0.01) |
| health | 0.23*** | 0.28*** | 0.27*** | 0.19*** |
|  | (0.03) | (0.05) | (0.03) | (0.03) |
| sex | -0.24*** | -0.03 | 0.04 | -0.24*** |
|  | (0.02) | (0.03) | (0.02) | (0.02) |
| account | -0.06** | 0.03 | 0.04 | -0.06** |
|  | (0.02) | (0.04) | (0.02) | (0.02) |
| age | 0.00 | -0.005*** | -0.001 | 0.00 |
|  | (0.001) | (0.002) | (0.001) | (0.001) |
| educ | 0.11*** | 0.41*** | -0.09*** | 0.09*** |
|  | (0.02) | (0.04) | (0.02) | (0.02) |
| familysize | -0.01* | -0.01 | 0.02*** | -0.01** |
|  | (0.004) | (0.01) | (0.01) | (0.004) |
| lnincome | 0.04*** | 0.08*** | 0.02 | 0.03** |
|  | (0.01) | (0.02) | (0.01) | (0.01) |
| nm | -0.24*** | -0.37*** | -0.34*** | -0.18*** |
|  | (0.02) | (0.04) | (0.02) | (0.02) |
| sc |  |  |  | 0.07*** |
|  |  |  |  | (0.01) |
| pc |  |  |  | 0.12*** |
|  |  |  |  | (0.01) |
| R^2 | 0.08 | 0.07 | 0.06 | 0.11 |
| Adj. R^2 | 0.08 | 0.07 | 0.06 | 0.11 |

*p<0.1; **p<0.05; ***p<0.01. pm, positive component of mental health;nm, negative component of mental health;pc, psychological capital; sc,social capital; educ, education; health, physical health.

**TABLE 5 |** Indirect path: "nm" (X) ==> "sc" (M) ==> "worksatisfy" (Y)

|  | Effect | S.E | z | p | [Boot 95% CI] |
| --- | --- | --- | --- | --- | --- |
| Indirect (ab) | -0.027 | 0.004 | -7.289 | <0.001*** | [-0.035, -0.020] |
| Direct (c') | -0.176 | 0.022 | -7.925 | <0.001*** | [-0.220, -0.132] |

**TABLE 6 |** Indirect path: "nm" (X) ==> "pc" (M) ==> "worksatisfy" (Y)

|  | Effect | S.E | z | p | [Boot 95% CI] |
| --- | --- | --- | --- | --- | --- |
| Indirect (ab) | -0.036 | 0.005 | -7.611 | <0.001*** | [-0.046, -0.027] |
| Direct (c') | -0.176 | 0.022 | -7.925 | <0.001*** | [-0.220, -0.132] |

**Robustness Analysis**

**TABLE R1 |** The mental health regressed on job satisfaction.

|  | （1） | （2） | （3） | （4） | （5） |
| --- | --- | --- | --- | --- | --- |
| mental |  | -0.29*** | -0.26*** | -0.24*** | -0.21*** |
|  |  | (0.01) | (0.02) | (0.02) | (0.01) |
| sc |  |  | 0.08*** |  | 0.07*** |
|  |  |  | (0.01) |  | (0.01) |
| pc |  |  |  | 0.11*** | 0.10*** |
|  |  |  |  | (0.01) | (0.01) |
| health | 0.28*** | 0.21*** | 0.19*** | 0.18*** | 0.17*** |
|  | (0.03) | (0.03) | (0.03) | (0.03) | (0.03) |
| sex | -0.23*** | -0.24*** | -0.24*** | -0.25*** | -0.24*** |
|  | (0.02) | (0.02) | (0.02) | (0.02) | (0.02) |
| account | -0.08*** | -0.07*** | -0.07*** | -0.07** | -0.07*** |
|  | (0.02) | (0.02) | (0.02) | (0.02) | (0.02) |
| marriage | 0.01 | 0.002 | 0.01 | -0.01 | 0.003 |
|  | (0.03) | (0.03) | (0.02) | (0.02) | (0.02) |
| age | -0.05*** | -0.05*** | -0.04*** | -0.05*** | -0.04*** |
|  | (0.01) | (0.01) | (0.01) | (0.01) | (0.01) |
| age^2 | 0.001*** | 0.001*** | 0.001*** | 0.001*** | 0.001*** |
|  | (0.0001) | (0.0001) | (0.0001) | (0.0001) | (0.0001) |
| educ | 0.13*** | 0.11*** | 0.08*** | 0.12*** | 0.09*** |
|  | (0.02) | (0.02) | (0.02) | (0.02) | (0.02) |
| familysize | -0.003 | -0.01* | -0.01 | -0.01** | -0.01** |
|  | (0.004) | (0.004) | (0.004) | (0.004) | (0.004) |
| lnincome | 0.07*** | 0.06*** | 0.05*** | 0.06*** | 0.05*** |
|  | (0.01) | (0.01) | (0.01) | (0.01) | (0.01) |
| Constant | 3.65*** | 4.25*** | 3.82*** | 3.71*** | 3.40*** |
|  | (0.15) | (0.15) | (0.16) | (0.16) | (0.16) |
| N | 6741 | 6741 | 6741 | 6741 | 6741 |
| R^2^ | 0.07 | 0.10 | 0.11 | 0.11 | 0.13 |

Robust standard errors in parentheses, *p<0.1; **p<0.05; ***p<0.01. mental, mental health; pc, psychological capital; sc,social capital; educ, education; health, physical health.

**TABLE R2 |** The results of instrumental regression.

| Dependent variable | Job satisfaction | |
| --- | --- | --- |
|  | (1) | (2) |
| mental | -2.08*** | -1.81** |
|  | (0.48) | (0.52) |
| health |  | -0.16 |
|  |  | (0.13) |
| sex |  | -0.28*** |
|  |  | (0.03) |
| account |  | -0.01 |
|  |  | (0.03) |
| marriage |  | -0.11** |
|  |  | (0.03) |
| age |  | 0.001 |
|  |  | (0.002) |
| educ |  | -0.0003 |
|  |  | (0.04) |
| familysize |  | -0.03*** |
|  |  | (0.01) |
| lnincome |  | 0.01 |
|  |  | (0.02) |
| Constant | 7.03*** | 6.92*** |
|  | (0.79) | (1.17) |
| N | 6741 | 6741 |
| Adj R^2^ | -0.97 | -0.63 |

Robust standard errors in parentheses, *p<0.1; **p<0.05; ***p<0.01. mental, mental health; educ, education; health, physical health.

**Mechanism Analysis**

**TABLE R3 |** The method of regression

|  | (1) worksatisfy | (2) sc | (3) pc | (4) worksatisfy |
| --- | --- | --- | --- | --- |
| (intercept) | 3.57*** | 5.55*** | 4.16*** | 3.57*** |
|  | (0.01) | (0.02) | (0.01) | (0.01) |
| health | 0.22*** | 0.24*** | 0.23*** | 0.18*** |
|  | (0.03) | (0.05) | (0.03) | (0.03) |
| sex | -0.23*** | -0.02 | 0.05* | -0.24*** |
|  | (0.02) | (0.03) | (0.02) | (0.02) |
| account | -0.05** | 0.03 | 0.05* | -0.06** |
|  | (0.02) | (0.04) | (0.02) | (0.02) |
| age | 0.00 | -0.006*** | -0.001 | 0.00 |
|  | (0.001) | (0.002) | (0.001) | (0.001) |
| educ | 0.11*** | 0.40*** | -0.10*** | 0.09*** |
|  | (0.02) | (0.04) | (0.02) | (0.02) |
| familysize | -0.01** | -0.01 | 0.02*** | -0.01** |
|  | (0.004) | (0.01) | (0.01) | (0.004) |
| lnincome | 0.04*** | 0.08*** | 0.01 | 0.03** |
|  | (0.01) | (0.02) | (0.01) | (0.01) |
| mental | -0.30*** | -0.51*** | -0.48*** | -0.22*** |
|  | (0.02) | (0.04) | (0.02) | (0.02) |
| sc |  |  |  | 0.07*** |
|  |  |  |  | (0.01) |
| pc |  |  |  | 0.10*** |
|  |  |  |  | (0.01) |
| N | 6741 | 6741 | 6741 | 6741 |
| Adj. R^2 | 0.08 | 0.07 | 0.06 | 0.11 |

*p<0.1; **p<0.05; ***p<0.01. mental, mental health; pc, psychological capital; sc,social capital; educ, education; health, physical health.

**TABLE R4 |** Indirect path: "mental" (X) ==> "sc" (M) ==> "worksatisfy" (Y)

|  | Effect | S.E | z | p | [Boot 95% CI] |
| --- | --- | --- | --- | --- | --- |
| Indirect (ab) | -0.036 | 0.004 | -8.078 | <0.001*** | [-0.044, -0.027] |
| Direct (c') | -0.217 | 0.023 | -9.475 | <0.001*** | [-0.260, -0.171] |

**TABLE R5 |** Indirect path: "mental" (X) ==> "pc" (M) ==> "worksatisfy" (Y)

|  | Effect | S.E | z | p | [Boot 95% CI] |
| --- | --- | --- | --- | --- | --- |
| Indirect (ab) | -0.047 | 0.006 | -7.751 | <0.001*** | [-0.060, -0.036] |
| Direct (c') | -0.217 | 0.023 | -9.475 | <0.001*** | [-0.260, -0.171] |
